# Prospective, randomized, controlled, open-label study to compare efficacy of a mineral-rich solution vs normal saline after complete ethmoidectomy

**DOI:** 10.1007/s00405-018-5232-9

**Published:** 2018-12-08

**Authors:** Ludovic de Gabory, Virginie Escabasse, Philippe Boudard, Guillaume de Bonnecaze, Cécile Rumeau, Roger Jankowski, Christian Debry, Sylvain Morinière, Bertrand Merino, Geoffrey Mortuaire, Olivier Malard, Laurence Bordenave

**Affiliations:** 1 ENT Department, CHU Bordeaux, 33000 Bordeaux, France; 2 CHU Bordeaux, CIC 14-01 IT, 33000 Bordeaux, France; 3 Univ. Bordeaux, 33000 Bordeaux, France; 4 ENT Department, Intercommunal Hospital of Créteil, Paris, France; 5 ENT Department, Saint-Augustin Private Hospital, Bordeaux, France; 6 ENT Department, University Hospital of Toulouse, Toulouse, France; 7 ENT Department, University Hospital of Nancy, Nancy, France; 8 ENT Department, University Hospital of Strasbourg, Strasbourg, France; 9 ENT Department, University Hospital of Tours, Tours, France; 10 Nuclear Medicine Department, Saint-Augustin Private Hospital, Bordeaux, France; 11 ENT Department, University Hospital of Lille, Lille, France; 12 ENT Department, University Hospital of Nantes, Nantes, France; 13 Nuclear Medicine Department, CHU Bordeaux, 33000 Bordeaux, France; 14 ENT Department, University Hospital of Bordeaux, Hôpital Pellegrin, Centre F-X Michelet, Place A. Raba-Léon, 33076 Bordeaux Cedex, France

**Keywords:** Nasal irrigation, Chronic rhinosinusitis, Nasal polyposis, Mucociliary clearance, Lund–Kennedy endoscopic score

## Abstract

**Purposes:**

The purpose of this study was to compare the efficacy of a mineral-rich solution vs normal saline solution (0.9% NaCl) following endoscopic complete bilateral ethmoidectomy.

**Methods:**

This was a prospective, multicenter, randomized, controlled, open-label trial in subjects suffering from steroid-resistant sinonasal polyposis. Adults performed 4 nasal irrigations of mineral or saline solutions daily for 28 days. Evaluations included subject-reported RHINO quality of life (QoL) and NOSE scores, tolerability, and satisfaction, the Lund–Kennedy endoscopic score and assessments of crusting, secretions and mucociliary clearance (rhinoscintigraphy).

**Results:**

A total of 189 subjects were randomized. Clinically relevant improvements (> 20 points) in RhinoQOL and NOSE scores were measured in both groups without any significant inter-group difference. Among the subjects with impaired RhinoQOL at pre-inclusion, the change in Impact-RhinoQOL score was significantly superior in mineral-rich vs saline solution at day 21 (*p* = 0.028) and day 28 (*p* = 0.027). The Lund–Kennedy score continuously improved in both groups earlier with the mineral-rich solution. Crusts were significantly fewer in number and less severe/obstructive in patients receiving mineral-rich vs saline solution at day 7 (*p* = 0.026) and day 14 (*p* = 0.016). Furthermore, secretions disappeared significantly more quickly and were less thick/purulent with mineral-rich solution at day 14 (*p* = 0.002) and day 21 (*p* = 0.043). Less epistaxis was reported in the mineral vs saline solution (*p* = 0.008 at day 21).

**Conclusions:**

Our findings indicate that the composition of a nasal irrigation solution influences endoscopic scores and QoL after sinus surgery for patients over 60, those with an initially poor QoL and higher symptom score, and smokers.

## Introduction

Nasal irrigation (NI) is one of the most important post-operative management strategies after endoscopic sinus surgery (ESS) [1–7]. It aims to cleanse the nasal cavities, promote wound healing, avoid local infection, and prevent future relapses [5]. Despite being recognized as important, recommendations concerning NI are lacking in terms of the specific method and composition of the solution to be used post-operatively. Indeed, there is little consensus about the efficacy of the numerous NI devices and solutions available [8, 9]. A recent Cochrane review highlighted the low quality and quantity of evidence regarding saline irrigation for chronic rhinosinusitis (CRS) [10].

The post-operative efficacy of nasal irrigations depends on several factors. Large-volume low-pressure nasal irrigation using undiluted seawater appears to be the best method [8]. Indeed, in vitro data are in favor of a solution rich in minerals compared to normal saline: calcium increases ciliary beat frequency (CBF) [11, 12], potassium is involved in epithelial repair and has an anti-inflammatory action [13, 14], magnesium increases the production of several growth factors, angiogenesis, and healing, decreases local inflammation and apoptosis [15], bicarbonates increase mucus quality [16] and finally sodium impairs CBF and healing due to competition with the calcium stimulus [17]. Others showed in vitro that a mineral-rich solution similar in composition to seawater reduced production of the chemokine interleukin-8 by activated human respiratory epithelial cells and was superior to normal saline in terms of improving CBF and accelerating epithelial wound repair [18, 19]. Moreover, a beneficial effect of the solution has already been shown in clinical studies concerning CRS treatment finding an effect of the mineral-rich solutions [20–22]. To date, however, robust data are lacking on the clinical impact of mineral-supplemented solutions used post-operatively.

The purpose of the present study was to compare the efficacy of mineral-rich solution similar to seawater vs saline solution (0.9% NaCl) during post-operative nasal irrigation in terms of quality of life and nasal tissue healing after surgical treatment for nasal polyposis.

## Materials and methods

### Study design

This study was conducted at nine French tertiary referral centers from April 2015 to March 2017. This was a prospective, randomized, controlled, open-label, single-blind, phase IV trial (Clinicaltrials.gov number NCT02559284) in patients receiving operations for steroid-resistant sinonasal polyposis.

The study duration was 28 days and visits were scheduled at pre-inclusion, days 7, 14, 21, and 28. The trial was approved by both the French National Health Agency and the regional ethics committee, and all procedures performed were in accordance with the ethical standards of the institutional research committee and with the Helsinki declaration. Written informed consent was obtained from all participants included in the study.

### Population, sample size, and randomization

Adult subjects aged 18–70 years suffering from steroid-resistant nasal polyposis requiring surgical treatment (bilateral and complete ethmoidectomy) were included. All phenotypes of nasal polyposis were included: an isolated sinonasal polyposis or a sinonasal polyposis associated with asthma and/or intolerance to nonsteroidal anti-inflammatory drugs (NSAIDS). Exclusion criteria included oral corticosteroid treatment in the previous 2 months, cystic fibrosis, anticoagulant use, uncontrolled diabetes, autoimmune diseases affecting the nose and/or sinuses, current or history of head/neck radiotherapy, and current or recent chemotherapy (previous 3 months). Women of childbearing potential had to use effective contraception to be enrolled in the study.

The operation was systematically a complete ethmoidectomy removing all the bony lamellae and mucosa within the labyrinth, with middle antrostomy. The mucosa of the lamina papyracea, the ethmoid roof and the lateral face of the middle turbinate was at best removed completely or almost completely. Only when the anatomical structure prevented access to the ethmoid or the sphenoid sinuses was the inferior half of the middle turbinate removed like a septal spur. The olfactory clefts were released from polyps and from possible adenomatoid hamartomas associated with polyposis and attached to the roof of the nasal cavity in the anterior part of the olfactory cleft. The sphenoidotomy was performed only if the CT-scan and/or endoscopy showed participation polyposis within the sphenoid cavities. No enlargement of the frontal ostium was performed. A sample size of 100 patients was planned per group, for a total of 200 patients with a view to showing an eight-point difference in the total RhinoQoL score between the two groups [23, 24]. Blocked randomization stratified by center was used to assign subjects to the treatment groups.

### Nasal irrigation solutions

The normal saline 0.9% (CDM Lavoisier, Paris, France) was a sterile, ready-to-use solution with pH < 7. It is the reference treatment reimbursed by the social security service usually prescribed in France by HCPs. The test solution (mineral-rich) is made of mineral salt powder in pre-dosed sachets, to be dissolved by study subjects in water. The mineral-rich solution was composed of 5 pre-dosed mineral salts: sodium chloride, potassium chloride, calcium chloride, magnesium chloride, and sodium bicarbonate in 4 g sachets (Respimer® Netiflow® sachets, Laboratoire de la Mer® SAS, Saint-Malo, France). Its composition has already been described [8]. One sachet reconstituted with 240 mL of mineral water dissolves instantly due to its hydrophilic formulation, representing an isotonic solution equivalent to 9 g/L of NaCl, preservative-free with a controlled pH between 7.6 and 8.4.

### Nasal irrigation method and education

During the first 4 post-operative weeks, 4 nasal irrigations (240 mL/wash–120 mL/side) were performed daily with the mineral-rich solution or the saline solution. The investigators were not aware of the nature of the product that was allocated to the study subjects thanks to a randomization code and the products were delivered by pharmacies from investigating centers directly to the patient without any intermediary. Each solution was distributed within the post-operative cavities with the same transparent, squeezable and washable device (straight, watertight nozzle) (Netiflow® device, Laboratoire de la Mer® SAS, Saint-Malo, France). The nasal irrigation procedure required washing both nostrils with 180 mL of solution distributed equally on each side to remove crusting and clotting, a gentle blowing of the nose, and rinsing both nostrils with the remaining 60 mL of solution distributed equally on each side (no nose-blowing). Detailed information concerning the purpose of nasal irrigation and how to use and clean the device was given to patients upon inclusion. The day after surgery, a specialized nurse educated the subjects, using a demonstration video as needed, in how to perform nasal irrigation. If the subject could not understand the procedure even after repeated training, they were excluded from the study. Debridement was forbidden and was reserved only if the patient suffered from resistant pains, purulence and/or complete, and irreversible nasal obstruction despite the nasal irrigations.

### Study end points

Quality of life and symptoms were assessed at pre-inclusion, days 7, 14, 21, and 28 using the patient-reported Rhinosinusitis Quality-of-Life Survey (RhinoQoL) and NOSE questionnaires [23, 24]. Compliance with study treatments, epistaxis frequency and patient satisfaction using a 100 mm Visual Analog Scale (VAS) were assessed at pre-inclusion, days 7, 14, 21, and 28. Compliance was evaluated in the following way: the patient had to fill in a compliance notebook (number of bottle/sachets used declared by the patient per day—number of washes performed per day). This patient notebook was sent once a week (D + 7, D + 14, D + 21, and D + 28) to the pharmacovigilance team.

The healing process of nasal mucosa within the operative field was evaluated using the Lund–Kennedy endoscopic score (20 points) at the same date. Complete healing was considered to have been achieved when the endoscopic score was ≤ 8 points. The mucociliary clearance was measured by rhinoscintigraphy before surgery and 14 and 21 days after. Rhinoscintigraphy was carried out with patients from two centers (University Hospital of Bordeaux, Saint-Augustin private clinic) to ensure test standardization, reproducibility, and availability of the gamma camera (Discovery 670, GE Medical Systems) [25, 26]. Control values were obtained from subjects in the per protocol population at pre-inclusion, which were on average 10.3 ± 1.3 mm/min for the mineral-rich solution group and 10.2 ± 1.8 mm/min in the saline solution group. Safety was assessed by the incidence of adverse events (AEs) throughout the study.

### Statistical analysis

Quantitative variables were described in terms of total number, average, standard deviation, and 95% confidence interval (CI) for the mean, median, and interquartile range (25–75%). Their normal distribution was verified using the Shapiro–Wilk test. For matched data (intra-group pre/post-op comparison), if the values were in conformity with the normal distribution, the Student’s *t* test was applicable. If not, the non-parametric Wilcoxon test was used. For non-matched data (inter-group pre/post-op comparison), if the values were in conformity with a normal distribution, Student’s *t* test was applicable. If not, the non-parametric Mann–Whitney test was used. Qualitative variables were described in terms of total number, percentage and CI, and the variables in both groups were compared using the χ^2^ test. All tests were conducted using the *p* value approach, with a power of 20% and significance set at an alpha level of 0.05. A univariate analysis was performed. Tests were conducted using SAS® software 9.2 or later versions. Post-hoc analyses were performed on subgroups including subjects with most impaired RhinoQOL at pre-inclusion (frequency score ≤ 66, bothersomeness score ≤ 70, and impact score ≥ 32), patients over 60 years of age, and smokers [23, 24].

## Results

### Subject disposition and demographics/clinical characteristics

A total of 189 patients were randomized, 95 to the mineral-rich solution group and 94 to the normal saline solution group. More than 91% of patients (86 cases) completed the study in both groups. The main reasons for study discontinuation were loss to follow-up and subject’s request (1 patient in the mineral group and 3 in the normal saline group). One subject in the mineral group discontinued the study owing to an unrelated AE.

Baseline characteristics were similar between the groups (Table 1). The study included more male than female patients in both groups. A large proportion of subjects had polyposis associated with asthma and/or hypersensitivity to NSAIDs. Approximately one-quarter of subjects were smokers, with an average of 12.3 and 11.4 cigarettes/day in each group (Table 1). Past smokers represented 20% and 32.9% of the mineral solution and normal saline groups, respectively.

**Table 1:** Demographics and baseline characteristics

	Mineral solution (N = 95)	Normal saline (N = 94)
Demographics		
Gender		
Male	64 (67.4%)	67 (71.3%)
Female	31 (32.6%)	27 (28.7%)
Age (years)		
Mean ± SD (min, max)	48.7 ± 11.1 (20, 69)	51.0 ± 11.9 (19, 76)
Baseline characteristics		
Type of polyposis		
Uncomplicated	56 (58.9%)	48 (51.1%)
Associated with asthma or hypersensitivity to NSAIDS	39 (41.1%)	46 (48.9%)
Smoker		
Yes *N* (%)	23 (24.2%)	27 (28.7%)
Years of smoking		
*N*	14	18
Mean ± SD (min, max)	20.6 ± 14.4 (0, 50)	23.9 ± 12.7 (2, 49)
Number of cigarettes smoked/day		
*N*	20	23
Mean ± SD	12.3 ± 7.1	11.4 ± 7.8
Past smoker		
Yes *N* (%)	19 (20.0%)	31 (32.9%)
Number of years since tobacco cessation		
Mean ± SD	11.0 ± 11.3	13.5 ± 10.1

### Patient-reported outcomes in intention-to-treat population: quality-of-life scores

As shown in Table 2, surgery plus large-volume nasal irrigation improved the scores on RhinoQOL and NOSE questionnaires. Improvements were similar between the groups and were clinically relevant, as they exceeded 20 points as early as day 14 for RhinoQOL and day 7 for NOSE scores. Regarding RhinoQOL evolution, improvements were statistically significant in frequency and bothersomeness as early as day 14 in both groups, and in impact as early as day 21. Regarding NOSE evolution, improvements were highly significant as early as day 14 in both groups (*p* < 0.001). All results from day 21 on showed highly significant improvements vs pre-inclusion scores (*p* < 0.001). However, throughout the post-operative period, no difference was observed between the groups.

**Table 2:** Intention-to-treat (ITT) population: evolution of RhinoQOL and NOSE

	Day 0		Day 7		Day 14		Day 21		Day 28	
	Mineral	NaCl	Mineral	NaCl	Mineral	NaCl	Mineral	NaCl	Mineral	NaCl
RHINOQOL SCORE										
Frequency	46.4 ± 25.6	47.3 ± 20.5	57.0 ± 21.4	61.0 ± 18.6	74.6 ± 14.4	73.8 ± 17.4	82.5 ± 14.5	81.7 ± 15.9	86.7 ± 12.9	85.2 ± 14.7
Change vs D0			9.4 ± 30.8	15 ± 22.8	27.4 ± 27.4*	27.3 ± 22.2**	36.5 ± 29.8***	35.6 ± 20.9***	39.7 ± 28.3***	39.6 ± 21.1***
Bothersomeness	51.4 ± 24.1	51.5 ± 21.8	62.3 ± 20.2	65.5 ± 21.3	77.8 ± 15.1	77.8 ± 17.4	86.5 ± 12.7	84.8 ± 16.8	88.8 ± 13.7	87.7 ± 15.7
Change vs D0			10.2 ± 27.9	15.8 ± 26.2	26.3 ± 26.6*	27.1 ± 23.7**	36.1 ± 25***	34.8 ± 21.6***	37.4 ± 26.2***	37.6 ± 23.4***
Impact	45.9 ± 25.3	45.6 ± 21.8	40.5 ± 23.3	38.0 ± 25.3	21.5 ± 19.9	20.8 ± 19.3	12.1 ± 13.3	12.9 ± 14.6	8.4 ± 12.0	9.4 ± 12.7
Change vs D0			− 5 ± 32.1	− 7.9 ± 27.6	− 24.8 ± 30.4	− 24.3 ± 24.6	− 34.9 ± 28.1***	− 32.1 ± 20.1***	− 37.9 ± 28.2***	− 36.1 ± 20.3***
NOSE SCORE	74.3 ± 28.1	72.2 ± 25.6	51.2 ± 32.5	46.7 ± 34.9	27.7 ± 26.8	28.8 ± 29.7	18.3 ± 21.9	18.6 ± 23.4	13.3 ± 19.8	15.2 ± 23.0
Change vs D0			− 22.6 ± 37.4	− 26.8 ± 38.9	− 45.9 ± 40.9***	− 44.8 ± 35.6***	− 55.8 ± 36.1***	− 54.4 ± 32.1***	− 61.2 ± 34.1***	− 58.6 ± 32.3***

Change vs pre-inclusion significantly > 20 points: *p < 0.05; **p < 0.01; ***p < 0.001; No significant inter-group difference in ITT population

### Subgroup analysis in populations with impaired RhinoQoL scores, age > 60 years and smokers

**Fig. 1:**
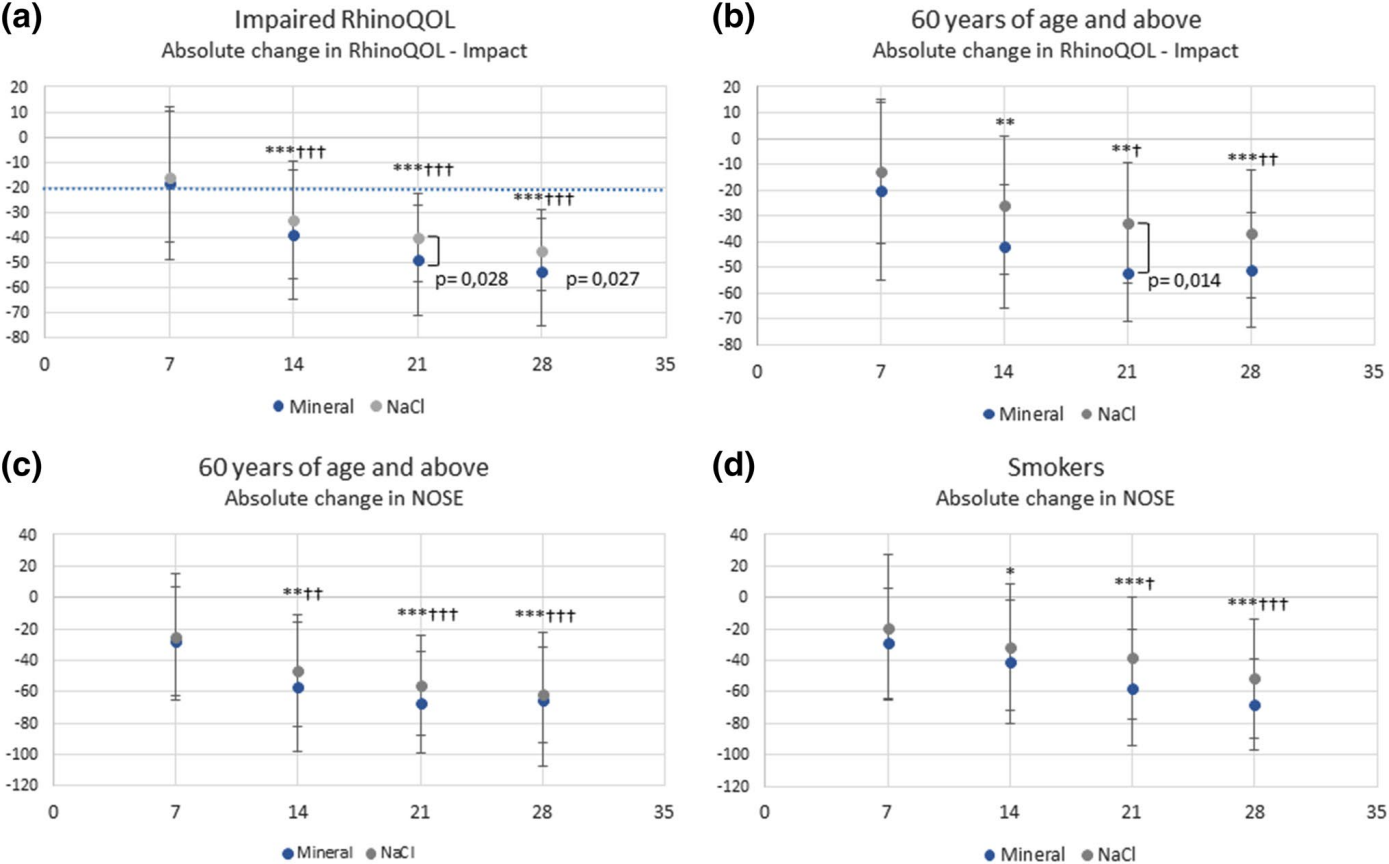
Subgroups with impaired cicatrization patterns: Change vs pre-inclusion in RhinoQOL Impact or NOSE score. *p < 0.05; **p < 0.01; ***p < 0.001 in mineral group: change vs pre-inclusion significantly > 20 points. †*p* < 0.05; ††*p* < 0.01; †††*p* < 0.001 in normal saline group: change vs pre-inclusion significantly > 20 points. Sample sizes: impaired RhinoQoL at pre-inclusion subgroup **a**: mineral: n = 52; normal saline: *n* = 55. > 60 year subgroup, **b, c**: mineral: *n* = 16; NaCl: *n* = 23. Smokers subgroup, **d** mineral: *n* = 23; NaCl: *n* = 27

In the most severely affected patients, i.e., those with impaired RhinoQOL at pre-inclusion, RhinoQOL impact scores improved significantly and clinically (> 20 points) vs pre-inclusion in both groups a week earlier compared to the ITT population, i.e., as of day 14 (Fig. 1a). A greater improvement was observed in the mineral group vs normal saline during the whole post-operative follow-up, reaching − 54.0 and − 45.3 points at day 28 in the mineral and normal saline groups, respectively. We observed significant differences in favor of the mineral solution vs normal saline on day 21 (*p* = 0.028) and day 28 (*p* = 0.027).

In patients over 60 years of age, improvements in RhinoQOL impact scores were clinically relevant as of day 7 in the mineral group vs day 14 in the normal saline group (Fig. 1b). These improvements were statistically significant as of day 14 in the mineral group and as of day 21 in the normal saline group. A greater improvement was observed in the mineral group vs normal saline during the whole post-operative follow-up, reaching − 50.9 and − 36.9 points at day 28 in the mineral and normal saline groups, respectively, and we observed a significant difference in favor of the mineral solution on day 21 (*p* = 0.014).

The mineral solution also provided greater and clinically relevant improvements of NOSE (> 20 points) in patients over 60 years during post-operative follow-up, but without reaching statistical difference between the groups (Fig. 1c).

Among smokers, clinically relevant improvements in NOSE score (> 20 points) were seen at day 7 in the mineral group and day 14 in normal saline group, respectively (Fig. 1d). These improvements were statistically significant vs pre-inclusion as of day 14 in the mineral group and day 21 in the normal saline group. The mineral solution provided a greater improvement in NOSE score during the entire post-operative follow-up, reaching − 68.3 points at day 28 vs − 51.3 points with normal saline, but without reaching statistical difference between the groups (Fig. 1d).

### Objective efficacy outcomes in ITT population—endoscopic parameters

**Fig. 2:**
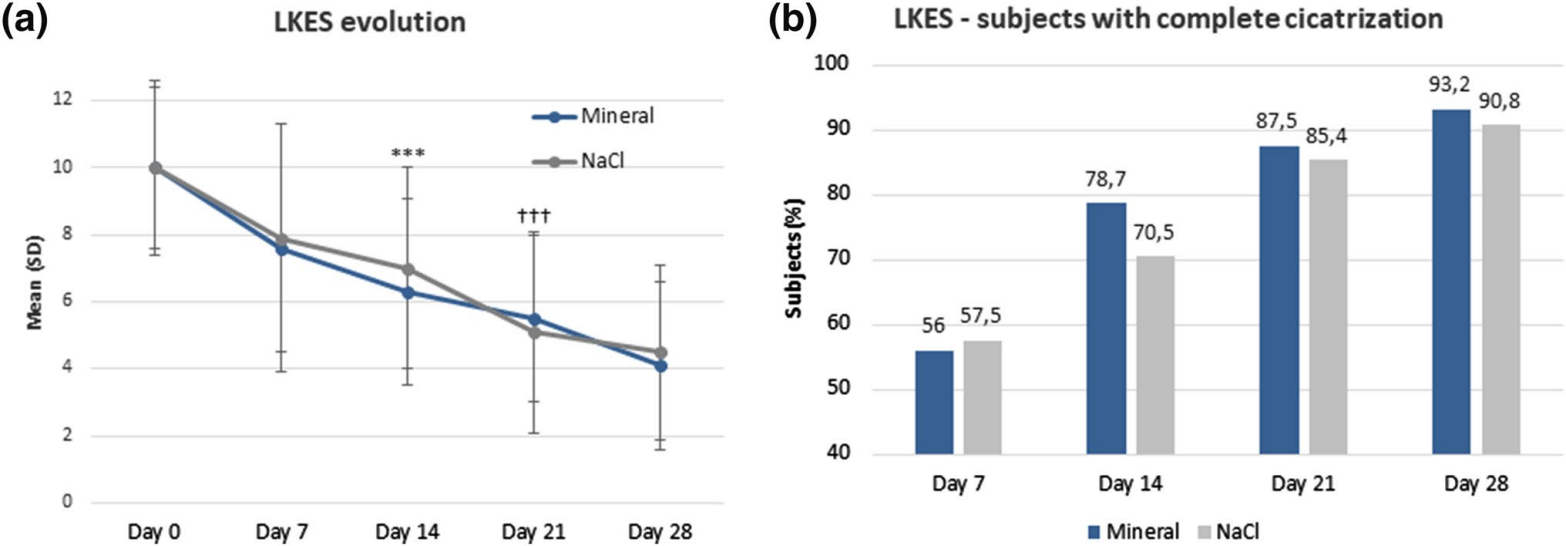
Lund–Kennedy endoscopic score: evolution and subjects (%) with complete cicatrization. ***Mineral group vs baseline, *p* ≤ 0.001; †††NaCl group vs baseline, *p* ≤ 0.001

**Fig. 3:**
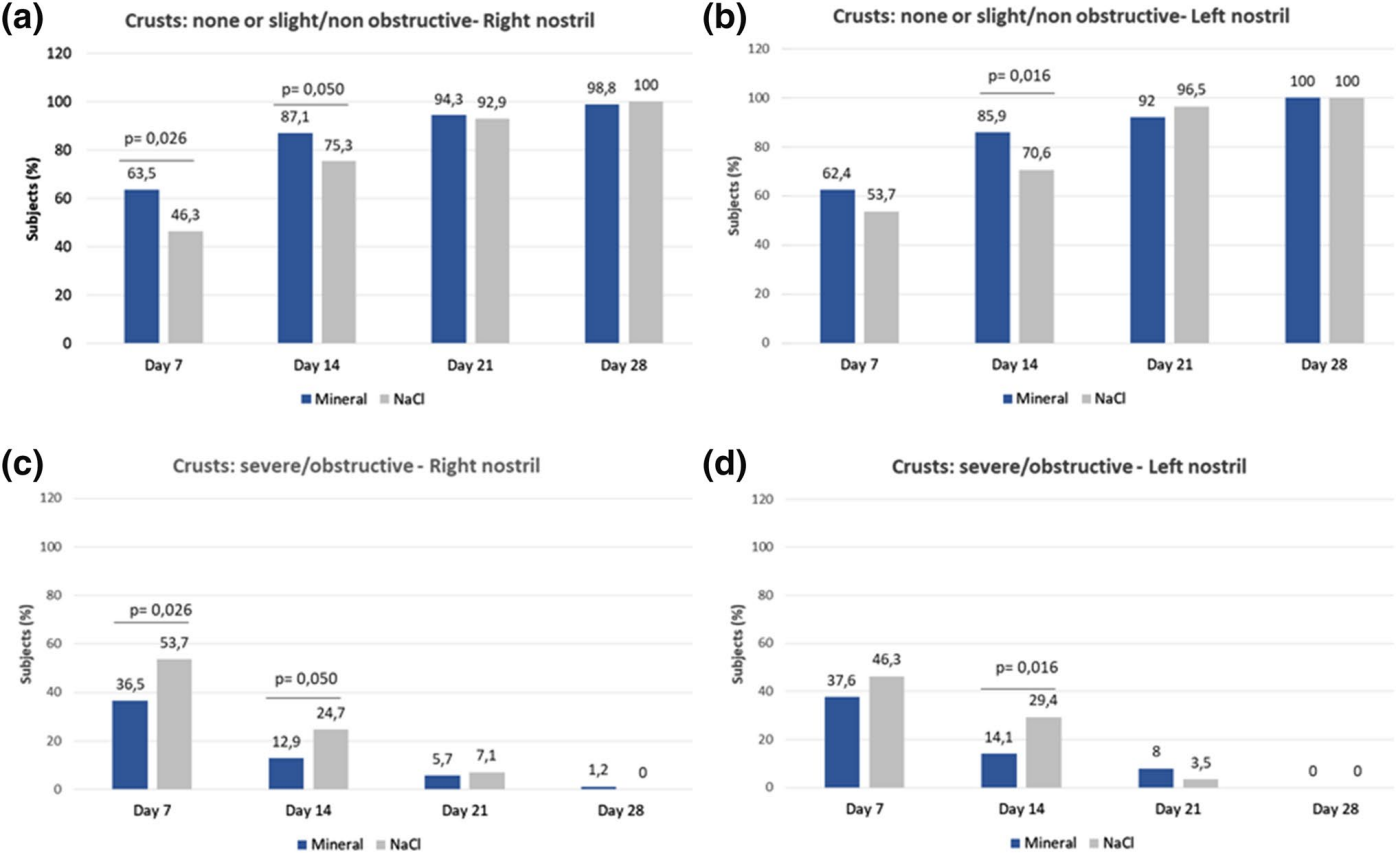
Lund–Kennedy endoscopic score: evolution of crusts

Continuous and robust improvement of the Lund–Kennedy score vs pre-inclusion occurred throughout the study in both groups (Fig. 2a). Improvement occurred significantly earlier in the mineral group vs normal saline as of day 14 compared to day 21, respectively (*p* ≤ 0.001). At day 28, although non-significant, a greater proportion of subjects reached a “near perfect” score (between 0 and 2) in the mineral group (35.2%) vs normal saline (27.6%). In line with these results, more subjects were found to have complete wound healing from day 14 in the mineral group, suggesting a faster resolution with the mineral-supplemented solution (Fig. 2b). In particular, crusts resolved significantly faster in the mineral group vs normal saline both at day 7 and day 14 (*p* ≤ 0.05, excluding the left nostril at day 7) (Fig. 3a, b). Furthermore, residual severe and obstructive crusts were significantly less frequent at day 7 and day 14 in the mineral group vs normal saline (*p* ≤ 0.05, excluding the left nostril at day 7) (Fig. 3c, d).

**Fig. 4:**
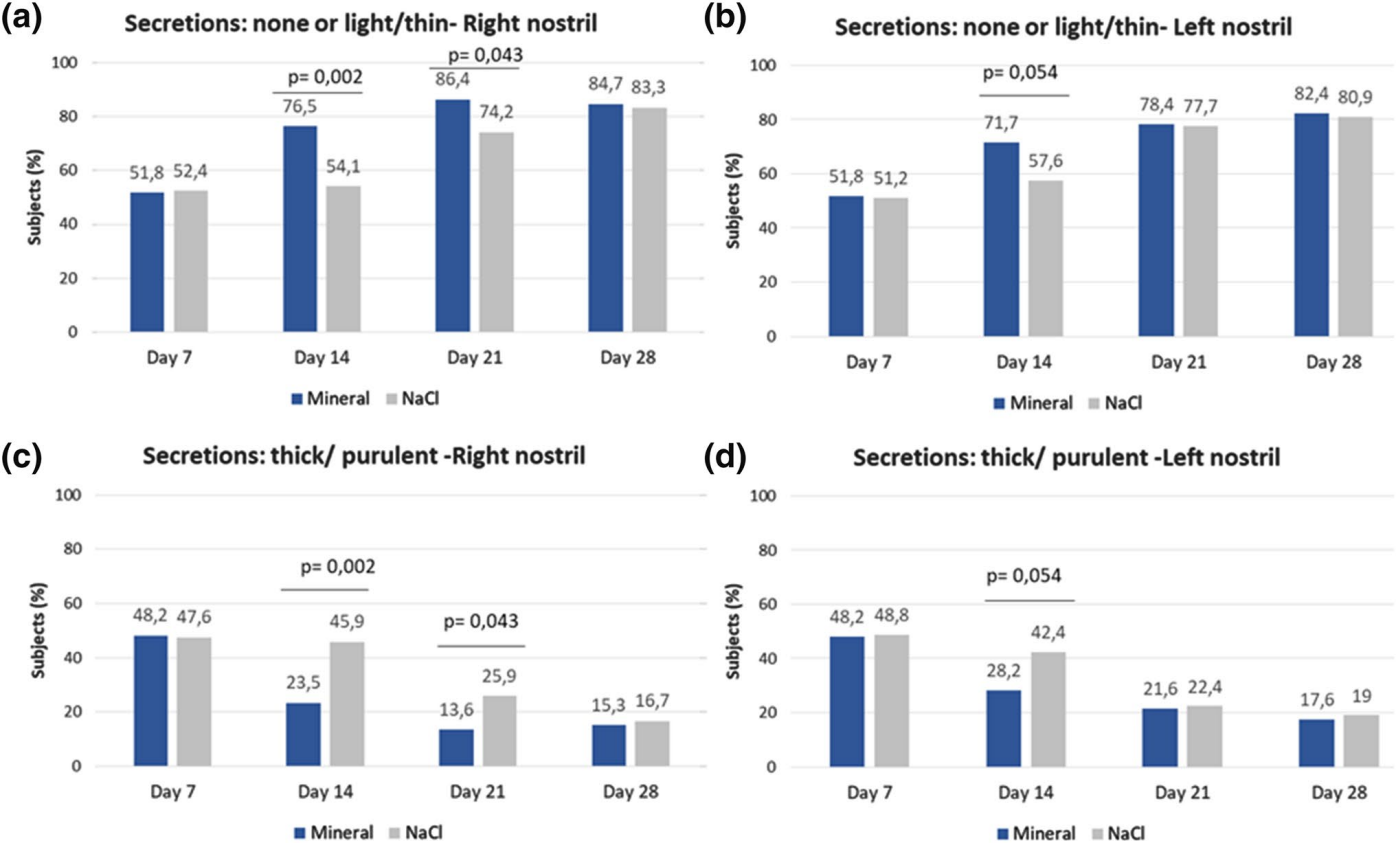
Lund–Kennedy endoscopic score: evolution of secretions

Like crusts, secretions also resolved significantly faster in the mineral group vs normal saline at day 14 and day 21 (*p* < 0.05 for the right nostril at both days and approaching significance (*p* = 0.054) for the left nostril at day 14; Fig. 4a, b). Furthermore, residual thick and purulent secretions were significantly less frequent at day 14 and day 21 in the mineral group vs normal saline (Fig. 4c, d). Matching the evolution of these key post-ESS endoscopic parameters, the intensity of epistaxis also decreased faster in the mineral group vs normal saline. This improvement was significantly greater at day 21 with the mineral solution (*p* = 0.008).

### Per protocol population: mucociliary clearance

**Fig. 5:**
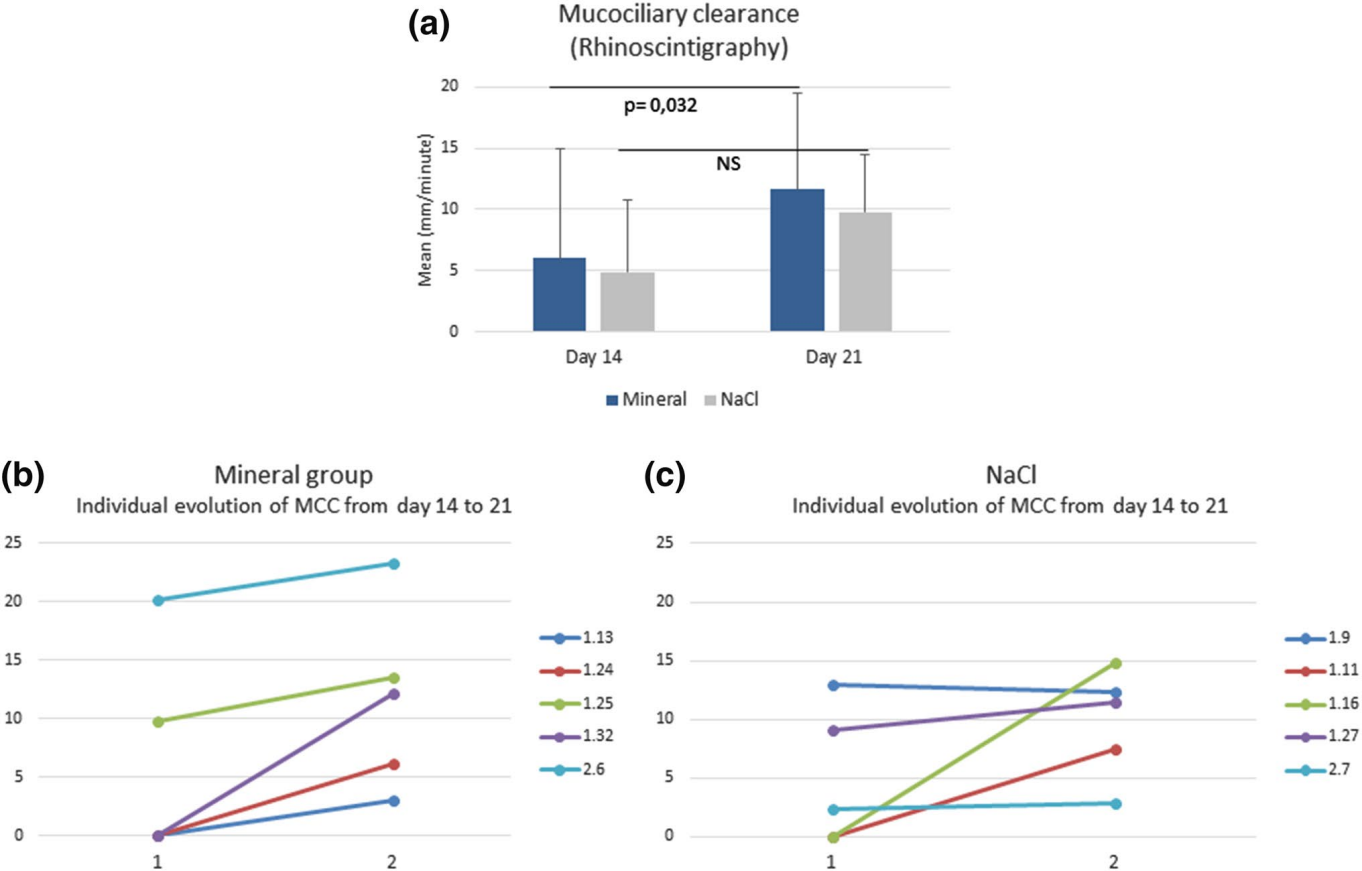
Mucociliary clearance measured by rhinoscintigraphy

Despite the small size of the sample, mucociliary clearance improved continuously with nasal irrigations performed with the mineral solution. Significant improvement was particularly notable from day 14 to day 21 (*p* = 0.032) vs normal saline (no significant change) (Fig. 5a). On average at day 21, rhinoscintigraphy values were 11.6 ± 7.8 mm/min and 9.8 ± 4.7 mm/min in the mineral and normal saline groups, respectively. Moreover, mucociliary clearance constantly improved in all subjects in the mineral group (Fig. 5b) vs normal saline (Fig. 5c).

### Compliance and satisfaction

Adherence to nasal irrigations (4 times/day) was sustained in both groups throughout the post-operative follow-up (average of 3.8 ± 0.15 and 3.8 ± 0.24 in the mineral and normal saline groups, respectively). Use of the device had increased the level of satisfaction by more than 90% at the end of the study. There was also a significant preference in terms of practicality in favor of the pre-dosed mineral salt sachets vs normal saline (*p* = 0.045). There was a strong willingness to continue nasal irrigation in both groups beyond the study period, reaching 89% and 82% of subjects in the mineral and normal saline groups, respectively.

### Safety outcomes

Only 1 subject (1.1% in the mineral group) discontinued the study early due to an unrelated AE. There were 6 and 5 serious AEs in the mineral and normal saline groups, respectively. One serious AE related to study or study procedure was present in each group: 1 subject with pain and crusts (mineral group) and 1 subject with headaches and crusts (normal saline group).

## Discussion

Large-volume low-pressure nasal irrigation rapidly improves QoL and endoscopic parameters of patients in the early post-operative weeks. This is the first randomized control study to directly compare the efficacy of two different solutions. Contrary to another study [27] and to avoid a major bias, nasal irrigation was delivered with the same device in both groups, because the volume, flow rate, and penetration angle of the solution have an effect on the efficacy of nasal irrigation, whatever the clinical context [8, 28].

Nasal irrigation shortly following ESS has been reported to promote wound healing and reduce nasal discharge and edema within the tissue [29–31], but evidence of the efficacy of various salt-based solutions in post-operative settings is lacking [32]. The present findings could impact post-operative care in several ways. The gradual improvement in symptom scores over 4 weeks was probably due to the combined action of the surgery, post-operative care, and the specific training in nasal irrigation. Rabago et al. found that a demonstration and coached practice in nasal irrigation resulted in effective and long-term care of patients with chronic sinonasal symptoms [33]. In our study, individualized training probably contributed to improving the quality of nasal irrigation and led to excellent adherence throughout the study.

Post-operative nasal irrigation in situation is not only useful for removing crusts, plots, secretions and cellular debris but also for promoting mucosal healing over a wide area of bare bone. Several in vitro studies have already demonstrated the potential for minerals and correct pH to promote the functional recovery of nasal epithelial cells [11, 14, 15, 19]. Moreover, in a clinical study by Low et al. Ringer lactate solution, another mineral-rich solution (pH 5.0–7.0) used to wash nasal cavities post-operatively, was more efficient in improving symptoms during the 6 weeks after surgery compared with normal saline and hypertonic saline solutions. However, in our study including the same post-operative period, changes in symptoms, QOL, endoscopic scores, and MCC were consistent and were in the same direction, whereas endoscopic and mucociliary clearance results were not concordant with symptom improvement in theirs [32].

In our study, the mineral-rich solution performed better, especially 7–10 days earlier. Clinically relevant results were observed from day 7 for the NOSE score and later for RhinoQoL scores in both groups, the difference with pre-inclusion scores becoming statistically significant after 21 days. The improvement in QoL scores at day 21 was greater than that obtained in the previous studies at day 28. These values are similar to those of an asymptomatic population in both groups [23, 24, 30, 31]. Our results are equivalent to the combined effect of high-volume irrigation plus nasal corticosteroid [31].

Considering the whole population, there was no statistical difference between both groups in our study. On the other hand, in the most severely affected patients such as those with high scores, a long-standing polyposis and/or smokers, there was a significant difference in efficacy in favor of the mineral-rich solution in terms of speed and intensity of recovery. Among subjects with an initially poor RhinoQoL score and smokers, the mineral solution was significantly superior regarding the impact on QOL. While data on this issue are sparse, a recent assay comparing tap water, buffered normal saline, buffered normal saline with xylitol, and hypertonic diluted seawater found that the best relief from nasal crusting, dryness, and obstruction following septoplasty and concha radiofrequency was obtained with hypertonic diluted seawater (*p* < 0.001) [27]. However, unlike in our study, smokers were not included, QoL was not measured, and mucociliary clearance times were not found to be different between groups [27].

Another finding in our study was that endoscopic scores improved to values twice as high as those previously reported, and higher than the effect of oral corticosteroids used before and after sinus surgery [34, 35]. Moreover, improvement in Lund–Kennedy endoscopic scores, healing of nasal mucosa, and reduction in epistaxis occurred more quickly with the mineral-rich solution. Both in the whole population and in subjects with an initially poor RhinoQoL score, there was a significantly faster reduction in secretions. Moreover, a significantly faster reduction in crusts occurred with the mineral-rich solution in the whole population and in smokers. A prospective study of the effect of smoking on Lund–Kennedy endoscopic scores and health-related QoL in 39 patients followed over 6 months after ESS found that the volume of daily smoking may worsen post-operative endoscopic scores [34]. Another study also showed a negative effect [35]. These results in favor of the mineral-rich solution are consistent with the above-mentioned in vitro data which attest to the anti-inflammatory healing characteristics of a mineral-rich solution with mild alkaline pH, leading to an improvement in ciliary beat frequency [11, 19]. Of course, it would be interesting to compare saline + buffer vs sea water, but this was not the objective of this study. Future work is needed, but in vitro we showed previously a statistical difference between isotonic and undiluted sea-derived saline vs isotonic, 2/3 diluted sea-derived saline, two solutions with a pH around 7.5 [19].

A higher percentage of subjects reached a nearly perfect endoscopic score by the end of the study using the mineral-rich solution compared to normal saline, along with a more rapidly improved Lund–Kennedy endoscopic score. Schlosser et al. demonstrated that excellent post-operative endoscopic scores are an indicator of better control of CRS, with improved symptoms and reduced use of systemic medication [34]. Therefore, targeting a faster improvement in endoscopic status may directly impact patients’ QoL and reduce the use of medications such as steroids [34, 36]. Moreover, as we observed, certain populations including patients over 60 years who may have a poor QoL or long-standing polyposis and those with impaired QoL would benefit from using a mineral-rich solution.

Our findings contribute new knowledge to current medical practice. Recommendations regarding how to perform nasal irrigation are vague; other than that, it should be done until healing is achieved [5, 9]. Indeed, a recent Cochrane review highlighted the low quality and quantity of evidence (only 2 studies) regarding saline irrigation for CRS [10]. As the number of irrigations per day has not been specified, we speculate that 3 or 4 washes per day could become a post-operative standard, while other studies of patients undergoing ESS recommend 2 or 3 times daily [27, 32, 37, 38]. Clearly, nasal irrigation is effective if used with good adherence on a daily basis.

Initially, we wanted to do a double-blind study, but NaCl powder pre-dosed sachets are not marketed in France and are too difficult to produce due to the stability of the product, the constraints to conserve a dry powder, and the cost. We, nevertheless, set up actions to conduct this study as closely as possible to a double-blind design, because investigators were not aware of the nature of the product that was allocated to the study subjects thanks to a randomization code. The study products were not delivered by investigators. Pharmacies from investigating centers oversaw delivering the first part of the study product (equivalent to the first 3 days) directly to the patient without any intermediary. They were blanked and conditioned within identical outer boxes for both treatments to prevent both pharmacists and subjects from disclosing the nature of the solution. Next, the remnant study products were directly delivered at home by an independent carrier. Moreover, the same medical distribution device was used in both groups. This study is also limited in that we did not determine a threshold for RhinoQoL as an inclusion criterion, because we assumed that all subjects would have a poor QoL given their advanced stage of polyposis. However, the subgroup analysis of those with impaired RhinoQOL at pre-inclusion showed a statistically significant improvement in QoL with the mineral-rich solution that was superior to normal saline. Finally, the rhinoscintigraphy sample was small due to the fact that rhinoscintigraphy is not commonly used in daily practice to measure CBF and is not available in all centers. Moreover, it needs test standardization and the reproducibility and availability of the same gamma camera (brand, type) to ensure that results are homogeneous. Therefore, to avoid numerous biases, we decided to limit the rhinoscintigraphy to the Nuclear Medicine Departments of Bordeaux. Next, some patients refused due to the irradiation, the number of supplementary consultations, the distance from the hospital and home, the schedule and duration of rhinoscintigraphy (almost 1 h) and their professional schedule.

## Conclusion

In line with recent clinical and in vitro studies, our findings indicate that the composition of a nasal irrigation solution influences endoscopic scores and QoL after sinus surgery for patients over 60, those with an initially poor QoL, and smokers. Mineral-rich solution improved results 7–10 days earlier than saline solution.
